# Dye Localization Extended Segmentectomy vs. Lobectomy for Deep Intersegmental Early-Stage Lung Cancer

**DOI:** 10.3390/diagnostics16050650

**Published:** 2026-02-24

**Authors:** Wen-Yao Lee, Ting-Fang Kuo, Hsiao-Hung Lu, Yu-Sen Huang, Min-Shu Hsieh, Hsao-Hsun Hsu, Jin-Shing Chen

**Affiliations:** 1 Graduate Institute of Pathology, National Taiwan University College of Medicine, Taipei 100233, Taiwan; yao120903@gmail.com (W.-Y.L.); mshsieh065@gmail.com (M.-S.H.); 2 Division of Thoracic Surgery, Department of Surgery, Fu Jen Catholic University Hospital, New Taipei 243089, Taiwan; 3 Division of Thoracic Surgery, Department of Surgery, National Taiwan University Hospital and National Taiwan University College of Medicine, Taipei 100225, Taiwan; tfkuomd@gmail.com (T.-F.K.); ntuhsu@gmail.com (H.-H.H.); 4 Institute of Biomedical Engineering, National Taiwan University College of Medicine and College of Engineering, Taipei 100233, Taiwan; 5 Department of Surgery, Shin Kong Wu Ho-Su Memorial Hospital, Taipei 111045, Taiwan; 6 Department of Medical Imaging, National Taiwan University Hospital, Taipei 100225, Taiwan; yusen0814@gmail.com; 7 Department of Radiology, National Taiwan University College of Medicine, Taipei 100233, Taiwan; 8 Department of Pathology, National Taiwan University Hospital, Taipei 100225, Taiwan; 9 Department of Surgical Oncology, National Taiwan University Cancer Center, Taipei 106037, Taiwan

**Keywords:** extended segmentectomy, lobectomy, CT-guided dye localization, deep intersegmental nodule, minimally invasive thoracic surgery

## Abstract

**Background:** Computed tomography-guided dye localization facilitates extended segmentectomy with reliable oncologic margins for deep intersegmental early-stage lung cancer. This study evaluated perioperative and long-term outcomes in comparison with those of lobectomy. **Methods:** We retrospectively reviewed patients with early-stage lung adenocarcinoma ≤ 2 cm who underwent computed tomography-guided dye localization extended segmentectomy between 2013 and 2019 and compared them with those who underwent lobectomy between 2011 and 2016. After 1:1 propensity score matching based on demographic and clinical variables, 30 matched pairs were included in the analysis. **Results:** Compared with lobectomy, extended segmentectomy with computed tomography-guided dye localization was associated with shorter operative time (102 ± 34 vs. 181 ± 42 min, *p* < 0.001), less blood loss (0 [0–0] vs. 0 [0–62.5] mL, *p* < 0.001), shorter chest tube duration (1 [1–2] vs. 2 [2–3] d, *p* = 0.002), reduced hospital stay (3 [3–4] vs. 5 [4–6] d, *p* < 0.001), and smaller ipsilateral (10.4 [1.9–15.7] vs. 20.0 [10.0–26.2] %, *p* = 0.004) and total (1.3 [−3.5–6.4] vs. 6.5 [1.4–12.9] %, *p* = 0.022) lung volume reductions at 6 months. All patients achieved negative resection margins. Lymph node yield was lower in the segmentectomy group (*p* < 0.001); however, the 5-year overall and disease-free survival rates were comparable. **Conclusions:** Computed tomography-guided dye localization extended segmentectomy provides favorable perioperative and functional outcomes and achieves comparable oncologic control in selected patients with deep intersegmental early-stage lung adenocarcinoma, representing a potential alternative to lobectomy.

## 1. Introduction

The incidence of pulmonary nodules has increased with the widespread use of low-dose computed tomography (CT), leading to earlier lung cancer diagnosis [1]. Recent randomized controlled trials have demonstrated that sublobar resection offers oncologic outcomes equivalent to those of lobectomy for early-stage lung cancer, as long as adequate resection margins can be obtained [2,3,4,5]. For lesions located near the intersegmental plane, extended segmentectomy, in which an additional margin from the adjacent segment is obtained using a non-anatomical resection, can be considered [6,7,8,9,10,11]. However, segmentectomy is technically more demanding than lobectomy, and determining the optimal resection line for small, nonpalpable lesions located deep within the pulmonary parenchyma remains challenging.

We developed a CT-guided patent blue vital (PBV) dye localization system for pulmonary nodules and demonstrated its feasibility, safety, and accuracy [12,13,14]. The technique was initially used in wedge resections and then applied to determine the resection line for extended segmentectomy, showing satisfactory results [10]. Nevertheless, the overall surgical outcomes remain unclear. This study aimed to compare the perioperative and long-term outcomes of CT-guided dye localization extended segmentectomy and lobectomy for early-stage lung adenocarcinoma.

## 2. Materials and Methods

### 2.1. Ethical Statement

This study was approved by the Research Ethics Committee of the National Taiwan University Hospital, Taipei, Taiwan (202502124RINE, 7 March 2025) and was conducted in accordance with the ethical principles of medical research involving human subjects outlined in the Declaration of Helsinki (2024), as amended by the World Medical Association. The requirement for informed consent was waived due to the retrospective nature of this study.

### 2.2. Study Population

**Figure 1:**
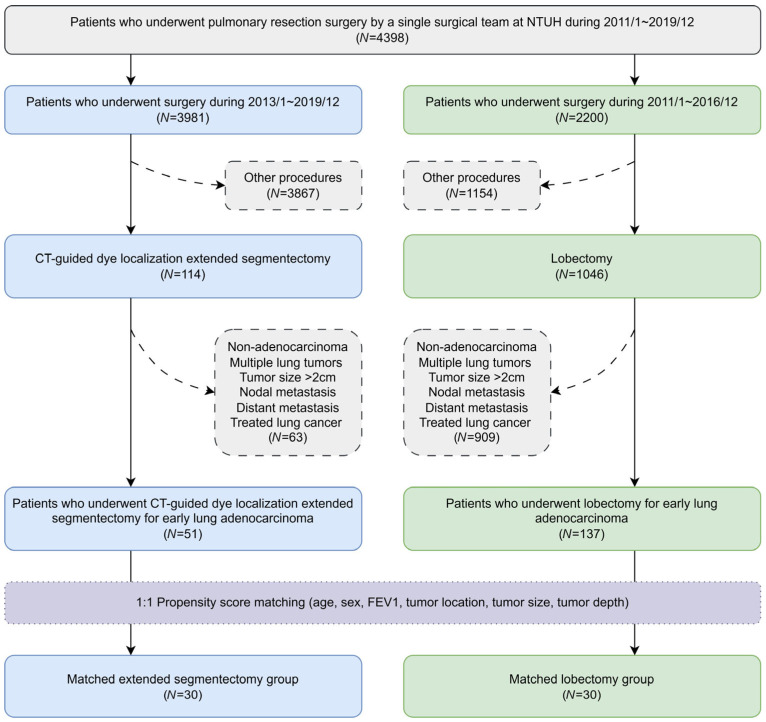
Flowchart of patient inclusion. CT: computed tomography; FEV_1_: forced expiratory volume in the first second; NTUH: National Taiwan University Hospital.

The flowchart of the patient inclusion process is shown in Figure 1. Patients who underwent pulmonary resection surgery by a single surgical team at the National Taiwan University Hospital between 1 January 2011 and 31 December 2019 were retrospectively reviewed. For stage IA lung cancer, segmentectomy was indicated for tumors ≤ 2 cm, as well as for tumors > 2 cm in patients with impaired cardiopulmonary function who were not considered suitable for lobectomy. Since early 2013, extended segmentectomy under CT-guided dye localization has been adopted for deep or intersegmental lesions in which adequate margins could not be confidently achieved using conventional segmentectomy. After excluding patients with non-adenocarcinoma, multiple lung tumors, tumor size > 2 cm, nodal metastasis, distant metastasis, or a history of treated lung cancer, 51 consecutive patients with early-stage lung adenocarcinoma who underwent CT-guided dye localization extended segmentectomy between 1 January 2013 and 31 December 2019 were included in the extended segmentectomy group. Similarly, another 137 patients with early-stage lung adenocarcinoma who underwent lobectomy between 1 January 2011 and 31 December 2016 were included in the lobectomy group.

### 2.3. Primary and Secondary Outcomes

The primary outcome was postoperative hospital stay. Secondary outcomes included perioperative outcomes (operative time, blood loss, chest tube duration, and resection margin) and long-term outcomes (lung volume reduction at 6 months, disease-free survival, and overall survival rates).

### 2.4. Surgical Techniques

**Figure 2:**
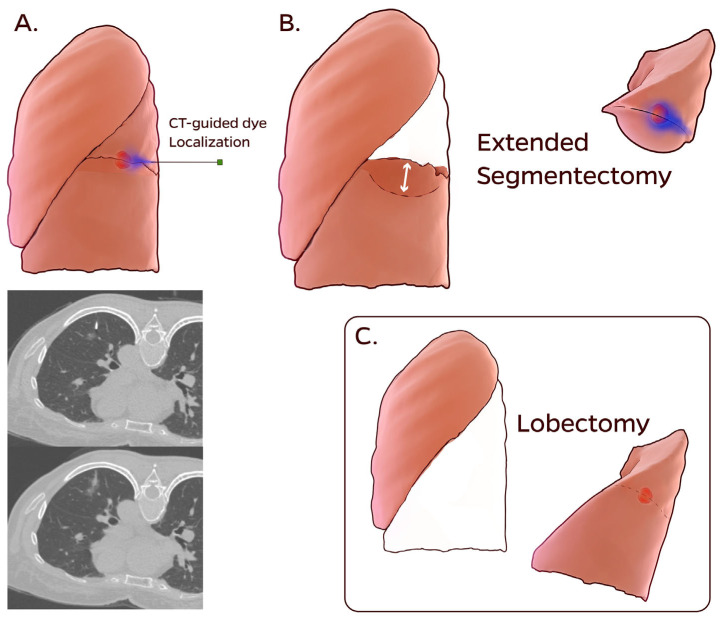
Illustration of CT-guided dye localization extended segmentectomy and lobectomy. (**A**) CT-guided localization of a nonpalpable pulmonary nodule located between left segment 6 and the basal segments. The upper CT image illustrates needle positioning during localization, whereas the lower CT image demonstrates the resulting dye distribution. (**B**) Dye localization guides the resection line beyond the virtual intersegmental plane to ensure adequate margins (double-headed arrow) during extended segmentectomy. (**C**) Lobectomy performed without dye localization. CT: computed tomography.

As shown in Figure 2 and Supplementary Video S1, the CT-guided dye localization extended segmentectomy technique has been previously described in detail [10,12,13,14].

The patients were transported to the CT suite on the day of the surgery. An initial CT scan was performed to confirm the most recent finding of a pulmonary nodule. Under local anesthesia and CT guidance, a 22-Gauge Chiba needle was advanced to the nodule or an adjacent site, and 0.1–0.3 mL of PBV dye (patent blue V 2.5%; Guerbet, Aulnay-sous-Bois, France) was injected into the nodule. For deep-seated nodules, when the dye was not visible on the pleural surface, an additional 0.2 mL injection was administered along the localization tract into the subpleural area. A repeat CT scan was then performed to evaluate the dye distribution and potential complications. The patients were subsequently transferred back to the ward to await surgery.

Extended segmentectomy was performed using video-assisted thoracoscopic surgery. Segmental arteries and veins were divided using curved-tip endovascular staplers (Johnson & Johnson Institute, Cincinnati, OH, USA), and the segmental bronchus and lung parenchyma were divided using endoscopic staplers (Johnson & Johnson Institute; Medtronic, Minneapolis, MN, USA). The dye markings and pinholes on the pleura guided tumor orientation, whereas the deeper dye facilitated precise localization. Together, these findings determined the resection line beyond the virtual intersegmental plane for extended segmentectomy to ensure adequate margins. Mediastinal lymph node dissection was performed in all patients.

### 2.5. Postoperative Management and Follow-Up

Intravenous analgesics were administered as required during the immediate postoperative period. Once patients resumed oral intake, additional analgesics, such as celecoxib and acetaminophen, were administered. Postoperative chest radiography was performed the following morning. The chest tube was removed when no air leak was detected and the drainage volume was <200 mL/d. Complications were classified using the extended Clavien–Dindo classification [15].

The endpoint of this study was all-cause mortality. Patients underwent CT and tumor-marker surveillance every 6 months for 5 years, and annually thereafter, until death or study termination (16 February 2025).

### 2.6. Data Collection and Statistical Analysis

Patient characteristics and perioperative and long-term outcomes were obtained from medical records. A five-factor modified Frailty Index (mFi-5) was used to assess frailty, which may influence postoperative outcomes. Preoperative and postoperative lung volumes were calculated from CT scans using AVIEW software version 1.1 (Coreline Soft, Seoul, Republic of Korea) [16].

Propensity score matching was used to balance baseline covariates, including age, sex, forced expiratory volume in the first second, and tumor location, size, and depth. The lobectomy group was 1:1 matched to the extended segmentectomy group using the greedy nearest-neighbor algorithm with a caliper of 0.02.

Statistical analyses were performed using SPSS Statistics (version 27.0; IBM, Armonk, NY, USA). Categorical data are presented as numbers (%) and were analyzed using Fisher’s exact or chi-square tests. Continuous data are presented as the median [interquartile range] or mean ± standard deviation. Normality was evaluated using the Shapiro–Wilk test. The Student’s *t*-test was used for normally distributed variables and the Mann–Whitney *U* test for non-normal distributions. Differences between the groups were reported using the Hodges–Lehmann estimator with 95% confidence intervals (CIs). Statistical significance was set at two-sided *p* < 0.05. Disease-free and overall survival were estimated using the Kaplan–Meier method and compared using the log-rank test.

## 3. Results

Patients in both groups were propensity score-matched at a 1:1 ratio, and 30 matched pairs were analyzed. The patient characteristics of the two matched groups are summarized in Table 1 and were comparable.

The perioperative outcomes are shown in Table 2. The median localization time for CT-guided dye localization extended segmentectomy was 19 min, with no localization-related complications greater than Clavien–Dindo grade II. The operative time was shorter (102 ± 34 vs. 181 ± 42 min, *p* < 0.001), and the blood loss was lower (0 [0–0] vs. 0 [0–62.5] mL, *p* < 0.001) in the extended segmentectomy group than in the lobectomy group. However, both the number of lymph node retrieval stations (3 [2–4] vs. 5 [5–6], *p* < 0.001) and the total number of lymph nodes retrieved (4 [3–8] vs. 12 [8–15], *p* < 0.001) were lower in the extended segmentectomy group than in the lobectomy group. Postoperatively, the extended segmentectomy group had a shorter chest tube duration (1 [1–2] vs. 2 [2–3] d, *p* = 0.002) and shorter postoperative hospital stay (3 [3–4] vs. 5 [4–6] d, *p* < 0.001). The resection margins were negative in both groups. The median pathological resection margin in the extended segmentectomy group was 1.0 cm. The 90-d morbidity rate did not differ significantly between the groups, and no 90-d mortality was observed.

The functional outcomes are summarized in Table 3. At 6 months, both ipsilateral and total lung volume reduction were significantly smaller in the extended segmentectomy group than in the lobectomy group (10.4 [1.9–15.7] vs. 20.0 [10.0–26.2] %, *p* = 0.004; and 1.3 [−3.5–6.4] vs. 6.5 [1.4–12.9] %, *p* = 0.022, respectively).

**Figure 3:**
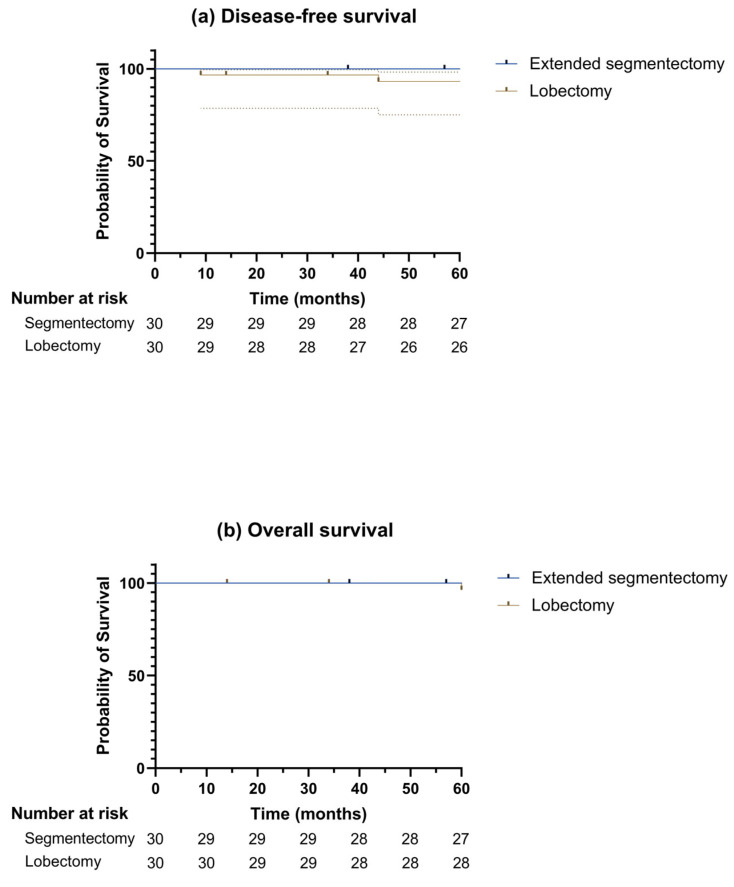
Kaplan–Meier Curves for (**a**) disease-free survival and (**b**) overall survival in patients with early-stage lung adenocarcinoma with tumor size ≤ 2 cm after lung resection surgery; the analysis between extended segmentectomy and lobectomy groups showed comparable long-term outcomes (*p* = 0.539 and 0.837 by log-rank test for disease-free survival and overall survival rates, respectively). Extended segmentectomy group, *N* = 30; lobectomy group, *N* = 30.

Figure 3 shows the overall and disease-free survival. The 5-year disease-free survival rates were 100% and 93.1% in the extended segmentectomy and lobectomy groups, respectively (*p* = 0.539). The 5-year overall survival rates were 100% and 96.4%, respectively (*p* = 0.837).

## 4. Discussion

CT-guided dye localization extended segmentectomy achieved shorter operative time, less blood loss, and shorter chest tube duration and hospital stay than lobectomy for deep intersegmental early-stage lung adenocarcinoma, with smaller lung volume reduction and comparable oncologic outcomes.

The non-inferiority of segmentectomy to lobectomy for stage IA1 and IA2 non-small cell lung cancer has been demonstrated in recent randomized trials [2,3,4,5]. Most of these studies included patients with peripheral lung lesions who underwent conventional segmentectomy to obtain adequate resection margins. However, segmentectomy for deep-seated nodules without compromising oncologic outcomes has also been reported in observational studies [17,18,19,20,21]. In real-world practice, intersegmental plane lesions are not uncommon, and determining the optimal parenchymal resection line for such lesions remains challenging, particularly when they are located deep within the pulmonary parenchyma [22]. Extended segmentectomy for these lesions has been facilitated in some centers by virtual-assisted lung mapping, intraoperative cone-beam CT-guided dye localization, or electromagnetic navigation bronchoscopy-guided radiofrequency identification marker placement to ensure adequate resection margins [8,9,10,11]. In addition to other emerging localization techniques, we developed a CT-guided dye localization method performed under local anesthesia in the CT suite to mark both the pleural and deep sites of the lesions. During extended segmentectomy, the inflation-deflation method and visible dyes delineate the resection line beyond the virtual intersegmental plane, ensuring adequate margins. Compared with other approaches, our cohort demonstrated short localization and operative times, and a 100% complete resection rate, indicating that the technique is simple yet effective [10].

The efficacy of new technology-assisted extended segmentectomy for deep intersegmental early-stage lung cancer, in terms of perioperative and long-term outcomes compared with lobectomy, has not been previously reported. Similar to prior studies comparing segmentectomy and lobectomy for small lung nodules, the 5-year overall and disease-free survival rates were comparable between the groups [2,3,4,5]. Although perioperative outcomes vary across the literature, most reports have shown that more lymph nodes are harvested in lobectomies than in segmentectomies, which is consistent with our findings. In contrast, our study demonstrated less blood loss and shorter operative time in the extended segmentectomy group, whereas previous reports have described comparable blood loss and longer operative times in the segmentectomy group. Chest tube duration and hospital stay were also shorter after extended segmentectomy than after lobectomy in our study, whereas they were generally comparable in a prior series. This may be attributed to the accurate CT-guided localization and stapler division of the intersegmental planes. Postoperative morbidity and mortality rates were not significantly different between the two groups, consistent with earlier reports [5,23,24,25,26]. We used CT-derived lung volume reduction as a surrogate for postoperative pulmonary function, as spirometric assessments were not routinely performed, particularly during the pandemic period [27,28]. At 6 months, both total and ipsilateral lung volume reductions were smaller in the extended segmentectomy group than in the lobectomy group, indicating that despite a slightly larger resection than conventional segmentectomy, extended segmentectomy effectively preserved lung function.

Our study had several limitations. First, the sample size in both groups was relatively small, which may have limited statistical power. Second, this was a retrospective study with comparisons based on historical controls. Although propensity score matching was applied to minimize selection bias and confounding factors, unmeasured or time-varying confounders may still exist. Finally, data on resection margins were incomplete due to the retrospective design; however, all available specimens had negative margins, and no local recurrence occurred in the extended segmentectomy group during follow-up.

## 5. Conclusions

CT-guided dye localization extended segmentectomy provides favorable perioperative and functional outcomes and achieves comparable oncologic control in selected patients with deep intersegmental early-stage lung adenocarcinoma, representing a potential alternative to lobectomy.

## Figures and Tables

**Table 1:** Patient Characteristics.

Variable	Extended Segmentectomy (*N* = 30)	Lobectomy (*N* = 30)	*p*-Value
Sex			1.000 ^b^
Male	10 (33)	11 (37)	
Female	20 (67)	19 (63)	
Age (years)	58 ± 12	59 ± 9	0.744 ^c^
BMI (kg/m^2^)	23.1 ± 2.9	24.4 ± 3.0	0.079 ^c^
Past or current smoker	5 (17)	4 (13)	1.000 ^b^
Lung function			
FEV_1_	2.4 ± 0.5	2.3 ± 0.5	0.562 ^c^
FEV_1_ (% of predicted)	107 ± 11	108 ± 15	0.681 ^c^
FVC (% of predicted)	107 ± 11	108 ± 14	0.648 ^c^
mFi-5 ^a^			0.782 ^d^
0	19 (63)	21 (70)	
1	9 (30)	8 (27)	
2	2 (7)	1 (3)	
Clinical tumor size (cm)	1.2 ± 0.3	1.2 ± 0.4	0.534 ^c^
Consolidation-tumor ratio			0.589 ^b^
≤0.5	21 (70)	18 (60)	
>0.5	9 (30)	12 (40)	
Tumor site			0.679 ^d^
LUL	6 (20)	5 (17)	
LLL	10 (34)	8 (27)	
RUL	9 (30)	10 (33)	
RML	1 (3)	4 (13)	
RLL	4 (13)	3 (10)	
Tumor depth	2.2 ± 0.9	2.0 ± 1.7	0.504 ^c^
Intersegmental plane lesion	27 (90)	26 (87)	1.000 ^b^
Pathological tumor size	1.1 ± 0.3	1.0 ± 0.3	0.755 ^c^
Pathological stage			0.397 ^d^
IA1	21 (70)	17 (57)	
IA2	9 (30)	12 (40)	
IB	0 (0)	1 (3)	

Continuous data are presented as the means ± SD. Categorical data are presented as numbers (%). ^a^ A surrogate for frailty that may influence outcomes after lung resection surgery. ^b^ Fisher’s exact test, ^c^ Student’s *t*-test, and ^d^ chi-squared test. BMI: body mass index; FEV1: forced expiratory volume in the first second; FVC: forced vital capacity; LLL: left lower lobe; LUL: left upper lobe; mFi-5: five-factor modified frailty index; RLL: right lower lobe; RML: right middle lobe; RUL: right upper lobe; SD: standard deviation.

**Table 2:** Perioperative Outcomes.

Variable	Extended Segmentectomy (*N* = 30)	Lobectomy (*N* = 30)	*p*-Value
Operative lobe or segment			0.679
LUL	6 (20)	5 (17)	
S1	1 (3)		
S2	1 (3)		
S1–3	3 (11)		
S4–5	1 (3)		
LLL	10 (34)	8 (27)	
S6	7 (24)		
S8	3 (10)		
RUL	9 (30)	10 (33)	
S2	2 (7)		
S3	7 (23)		
RML	1 (3)	4 (13)	
S5	1 (3)		
RLL	4 (13)	3 (10)	
S6	4 (13)		
Localization time (min)	19 (16–23)	NA	
Localization-related complications			
Pneumothorax	9 (30)	NA	
Intrapulmonary focal hemorrhage	14 (47)	NA	
Hemothorax	0 (0)	NA	
Complication > Clavien–Dindo II	0 (0)	NA	
Operative time (min)	102 ± 34	181 ± 42	<0.001
Blood loss (mL)	0 (0–0)	0 (0–62.5)	<0.001 ^a^
Lymph node stations retrieved	3 (2–4)	5 (5–6)	<0.001 ^b^
Lymph node yield	4 (3–8)	12 (8–15)	<0.001 ^c^
Time to chest tube removal (d)	1 (1–2)	2 (2–3)	0.002 ^d^
Postoperative hospital stays (d)	3 (3–4)	5 (4–6)	<0.001 ^e^
Pathological resection margin (cm)	1.0 ± 0.4	NA	
Positive resection margin	0	0	
90-d morbidity	0 (0)	4 (13)	0.112
Persistent air leaks	0 (0)	2 (7)	0.492
Bronchopleural fistula	0 (0)	0 (0)	
Pneumonia	0 (0)	0 (0)	
Arrhythmia	0 (0)	2 (7)	0.492
Morbidity > Clavien–Dindo II	0 (0)	1 (3)	1.000
90-d mortality	0 (0)	0 (0)	

Operative time and pathological resection margin are presented as the means ± SD, whereas other continuous data are presented as medians (IQR). Categorical data are presented as numbers (%). The *p*-values were calculated using Student’s *t*-test, Mann–Whitney *U* test, Fisher’s exact test, or chi-squared test, as appropriate. Differences between extended segmentectomy and lobectomy were reported using the Hodges–Lehmann estimator with 95% CIs. ^a^ Intergroup difference is 0 (95% CI: 0 to 0). ^b^ Intergroup difference is −2 (95% CI: −3 to −2). ^c^ Intergroup difference is −6 (95% CI: −9 to −4). ^d^ Intergroup difference is −1 (95% CI: −1 to 0). ^e^ Intergroup difference is −1 (95% CI: −2 to −1). CIs: confidence intervals; IQR: interquartile range; LLL: left lower lobe; LUL: left upper lobe; mFi-5: five-factor modified frailty index; NA: not available; RLL: right lower lobe; RML: right middle lobe; RUL: right upper lobe; S: segment; SD: standard deviation.

**Table 3:** Functional Outcomes.

Variable	Extended Segmentectomy (*N* = 30)	Lobectomy (*N* = 30)	*p*-Value
Preoperative CT lung volume			
Ipsilateral (mL)	1996 ± 480	2038 ± 526	0.749
Total (mL)	4026 ± 985	4088 ± 992	0.809
Postoperative CT lung volume			
Ipsilateral at 6 months (mL)	1843 ± 445	1677 ± 501	0.184
Total at 6 months (mL)	4000 ± 938	3861 ± 712	0.523
Lung volume reduction ^a^			
Ipsilateral (%)	10.4 (1.9–15.7)	20.0 (10.0–26.2)	0.004 ^b^
Total (%)	1.3 (−3.5–6.4)	6.5 (1.4–12.9)	0.022 ^c^

Total and ipsilateral lung volume reduction are presented as the median (IQR), whereas other continuous data are presented as the mean ± SD. *p*-values were calculated using Student’s *t*-test or Mann–Whitney *U* test, as appropriate. Differences between extended segmentectomy and lobectomy were reported using the Hodges–Lehmann estimator with 95% CIs. ^a^ Lung volume reduction was calculated from 1 mm CT scans. Patients without either pre- or postoperative 1 mm CT data (9 and 8 in the extended segmentectomy and lobectomy groups, respectively) were excluded from this analysis. ^b^ Intergroup difference is −10.5 (95% CI: −3.4 to −18.8). ^c^ Intergroup difference is −5.5 (95% CI: −0.7 to −11.0). CIs: confidence intervals; CT: computed tomography; IQR: interquartile range; SD: standard deviation.

## Data Availability

The authors confirm that the data generated and analyzed during this study and the raw data are available from the corresponding author upon reasonable request.

## Supplementary Materials

The following supporting information can be downloaded at: https://www.mdpi.com/article/10.3390/diagnostics16050650/s1, Video S1: This video demonstrates the technique of CT-guided dye localization extended segmentectomy and briefly compares its perioperative and long-term outcomes with those of lobectomy.

## Abbreviations

CT	computed tomography
PBV	patent blue vital
mFi-5	five-factor modified Frailty Index
CIs	confidence intervals
